# Fusion to a homo-oligomeric scaffold allows cryo-EM analysis of a small protein

**DOI:** 10.1038/srep30909

**Published:** 2016-08-03

**Authors:** Francesca Coscia, Leandro F. Estrozi, Fabienne Hans, Hélène Malet, Marjolaine Noirclerc-Savoye, Guy Schoehn, Carlo Petosa

**Affiliations:** 1 Institut de Biologie Structurale (IBS), Univ. Grenoble Alpes, CEA, CNRS, 38044 Grenoble, France

## Abstract

Recent technical advances have revolutionized the field of cryo-electron microscopy (cryo-EM). However, most monomeric proteins remain too small (<100 kDa) for cryo-EM analysis. To overcome this limitation, we explored a strategy whereby a monomeric target protein is genetically fused to a homo-oligomeric scaffold protein and the junction optimized to allow the target to adopt the scaffold symmetry, thereby generating a chimeric particle suitable for cryo-EM. To demonstrate the concept, we fused maltose-binding protein (MBP), a 40 kDa monomer, to glutamine synthetase, a dodecamer formed by two hexameric rings. Chimeric constructs with different junction lengths were screened by biophysical analysis and negative-stain EM. The optimal construct yielded a cryo-EM reconstruction that revealed the MBP structure at sub-nanometre resolution. These findings illustrate the feasibility of using homo-oligomeric scaffolds to enable cryo-EM analysis of monomeric proteins, paving the way for applying this strategy to challenging structures resistant to crystallographic and NMR analysis.

Recent technological advances have dramatically transformed single particle electron cryo-microscopy (cryo-EM), allowing 3D reconstructions of macromolecular complexes to be determined at near-atomic resolution^1^,^2^,^3^. While the earliest near-atomic resolution cryo-EM structures were limited to icosahedral viruses and high-symmetry complexes^4^,^5^, more recent structures involve macromolecular assemblies with low or no symmetry (e.g., refs 6, 7, 8, 9, 10, 11). Cryo-EM reconstructions have been reported at resolutions as high as 1.8–2.2 Å, allowing water molecules and cations to be identified and small-molecule ligands to be positioned with high accuracy^12^,^13^.

Besides improved resolution, advances in cryo-EM have also enabled the analysis of macromolecular species considerably smaller (<200 kDa) than was previously feasible. Selected examples include the tumour suppressor protein p53 (174 kDa)^14^, the mitochondrial outer membrane protein Tob55 (110 kDa)^15^, the heat-shock protein αB crystallin (121 kDa)^16^, the DNA-bound nuclear receptors RXR/VDR and USP/EcR (both ~100 kDa)^17^,^18^, the human γ-secretase complex (170 kDa)^8^,^19^ and isocitrate dehydrognenase (93 kDa)^13^. While many of these structures were determined at intermediate (6–17 Å) resolution, more recent studies have achieved near-atomic resolution (e.g., refs 13,19, 20, 21), further underscoring the rapid progress in the cryo-EM field. However, compared to the theoretical lower size limit of a protein whose structure can be determined by cryo-EM (20–40 kDa)^22^,^23^, the current practical limit is significantly higher (~100 kDa), as the small difference in density between protein and vitreous ice hampers the detection of particles and the determination of their orientations. Consequently, many macromolecular species, including most monomeric proteins of biomedical interest, are too small to be analyzed by cryo-EM.

Recent strategies devised to overcome this limitation include the use of specific monoclonal Fab fragments to increase the molecular mass of the target structure^24^,^25^, and flexibly attaching the target protein to a self-assembled DNA nanoaffinity template to facilitate imaging^26^. Here we describe an alternative approach, whereby a large, symmetric particle is generated by genetically fusing the monomeric target to a scaffold protein that self-assembles as a homo-oligomer. The approach is inspired by work in molecular vaccinology, in which sub-viral particles bearing capsid-incorporated epitopes serve as vaccine platforms to generate protective immunity^27^,^28^, and by studies in biomolecular design, in which rigid macromolecular assemblies (2D lattices and 3D cages) are constructed by fusing modules of known structure via carefully engineered junction residues^29^,^30^,^31^,^32^,^33^. A pioneering study in this area was performed with the hepatitis B virus (HBV) core protein, which spontaneously assembles into icosahedral capsid-like particles^28^. Kratz and co-workers engineered a chimeric version of the core protein in which green fluorescent protein (GFP, 27 kDa) was inserted within a loop on the HBV capsid surface. Cryo-EM analysis subsequently revealed an extra ring of density on the surface of the recombinant particle attributable to GFP. Because the GFP moiety was linked to the HBV core through flexible N- and C-terminal linkers, its structure was poorly ordered in the cryo-EM reconstruction following density averaging during the symmetry-constrained map calculation. Although efforts to improve the GFP structure were not pursued, it was proposed that optimizing the linkers could conceivably reduce GFP flexibility and lead to a better resolved structure in the cryo-EM map.

Here, we show that by fusing a monomeric protein to a homo-oligomeric scaffold and screening different-sized junction regions, a suitably rigid chimeric particle can be generated whose large size and high symmetry facilitates cryo-EM analysis, resulting in a 3D reconstruction of the target structure at sub-nanometre resolution. We anticipate that this strategy will prove a useful addition to the toolkit of methods available for investigating monomeric protein structures resistant to NMR and crystallographic analysis.

## Results

### Scaffolding strategy and selection of fusion proteins

**Figure 1:**
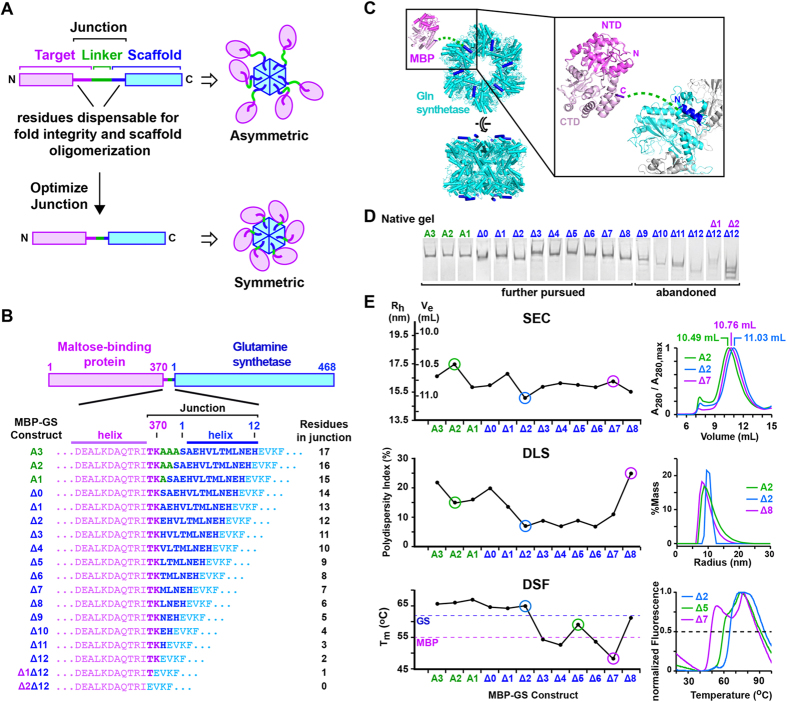
Scaffolding strategy and biophysical screening of MBP-GS fusion constructs. (**A**) Scaffolding strategy. *Top:* The target monomer (magenta) is fused via a linker (green) to the scaffold protein (blue). Boxes represent the minimal regions required for proper subunit folding and oligomer assembly. *Bottom:* The junction length is optimized to bring the structured domain(s) of the target into contact with the scaffold and thereby yield a rigid, symmetric particle. (**B**) MBP-GS fusion constructs and their junction sequences. (**C**) Ribbon diagram illustrating the fusion of MBP to GS. The MBP N-terminal domain (NTD) is in magenta and the C-terminal domain (CTD) in pink. The 2 C-terminal MBP and 12 N-terminal GS residues dispensable for fold stability and oligomerization are in purple and navy blue, respectively. (**D**) Native PAGE analysis of MBP-GS fusion constructs. (**E**) Biophysical analysis of constructs A3 to Δ8. The left panels summarize results for all 12 constructs. The right panels show raw data for 3 representative constructs (circled in green, blue and magenta) that illustrate the spread of values observed in each assay. *Top,* Summary of hydrodynamic radii (R_h_) determined by SEC, with representative chromatograms shown at right. *Middle,* Plot of polydispersity index determined by DLS, with profiles illustrated at right. *Bottom*. Summary of melting temperature (T_m_) determined by DSF, with representative thermal denaturation curves shown at right. See also Supplementary Figs S1–S3.

Our overall strategy is summarized in Fig. 1A. A chimeric protein is constructed by fusing the target monomer to a homo-oligomerizing scaffold protein via a heterologous linker long enough to allow proper folding of the fused subunits. Upon homo-oligomerization, the flexibility afforded by the linker and by any flanking disordered residues allows the target subunits to adopt different orientations relative to the scaffold, yielding an asymmetric chimeric particle (Fig. 1A, top). Subsequently, the number of connecting residues is optimized to induce the target subunits to adopt the scaffold symmetry. We define the “junction” as comprising the heterologous linker plus flanking target and scaffold residues dispensable for proper folding and oligomer assembly. (The maximal number of such flanking residues for a target protein of unknown structure can be determined empirically, e.g., by assessing the solubility and thermal stability of a C-terminal truncation series). The junction is optimized by progressively deleting linker and/or flanking residues to reduce the spatial separation between the two fusion partners, thereby restricting the relative mobility of the target subunits. In the ideal case, this procedure generates one or more constructs in which target subunits abut snugly against the scaffold in a uniform orientation, yielding a symmetric particle suitable for cryo-EM analysis (Fig. 1A, bottom).

To identify a suitable scaffold, we searched the Protein Data Bank (PDB) for proteins that formed large homo-oligomers and had a sterically accessible N- or C-terminus remote from the oligomerization interface, restricting our search to bacterial proteins so as to facilitate expression in *E. coli*. Negative stain EM analysis of three such proteins and examination of the literature led us to choose glutamine synthetase (GS) as an expedient scaffold (Supplementary Fig. S1A–C). GS forms a dodecamer with 6-fold dihedral (D_6_) symmetry and possesses a sterically accessible N-terminus. The first 12 residues of each monomer define an α helix which appears dispensable for subunit folding and oligomer stability, and can thus be partly or fully deleted during the junction-optimization step (Fig. 1B,C and Supplementary Fig. S1C).

For the purposes of this study we chose maltose-binding protein (MBP), a 40.7 kDa bacterial protein, as a convenient monomeric target, since MBP was well expressed when fused to GS (Supplementary Fig. S1D) and because its structure includes a mixture of features (α helices, β strands and a small-molecule ligand) which should aid visual assessment of the accuracy and resolution of cryo-EM maps. MBP is composed of two (N- and C-terminal) globular domains, each consisting of a central β-sheet flanked on both sides by 2 or 3 parallel helices (Fig. 1C). Inspection of the MBP crystal structure suggests that the two C-terminal residues (Thr369 and Lys370) can be deleted without compromising fold stability, whereas the preceding residue (Ile368), located at the end of an α-helix, packs within the hydrophobic core of the C-terminal domain. Crystal structures of MBP-fusion proteins show that a linker comprising as few as three alanine residues allows MBP to fold properly and to be flexibly connected to a C-terminal fusion partner. Accordingly, we designed our initial MBP-GS fusion protein to possess a tri-alanine linker (construct A3) and generated a series of deletion mutants in which we progressively removed 1–3 residues from the linker (constructs A2, A1 and Δ0), 1–12 residues from the N-terminus of GS (constructs Δ1-Δ12), and 1 or 2 C-terminal residues from MBP (constructs Δ1Δ12 and Δ2Δ12), yielding a panel of constructs with 0–17 residues in the junction (Fig. 1B).

### Biophysical screening and negative-stain EM of MBP-GS chimeras

We bacterially expressed the 18 MBP-GS fusion constructs and analyzed the purified proteins by native polyacrylamide gel electrophoresis (PAGE) to assess their ability to fold and assemble correctly (Fig. 1D). Whereas the longer constructs (A3-Δ8) predominantly migrated as relatively sharp, single bands, the six shortest constructs (Δ9-Δ2Δ12) migrated as multiple bands or displayed a smeared appearance, suggesting improper folding or incomplete oligomer assembly. We therefore abandoned these proteins and focused on the remaining 12 constructs (A3-Δ8), which we further characterized by size-exclusion chromatography (SEC), dynamic light scattering (DLS), and differential scanning fluorimetry (DSF). SEC analysis revealed that the hydrodynamic radius (R_h_) varied between 15.2 and 17.5 nm, fluctuating significantly for the six longest constructs (A3-Δ2) and adopting a more constant value for the shorter constructs (Δ3-Δ8) (Supplementary Fig. S2A and Fig. 1E, top). The smallest R_h_ value was observed for MBP-GSΔ2, indicating that this construct adopts the most compact conformation. The polydispersity index determined by DLS varied significantly (6.9–24.2%), and was highest for constructs A3-Δ0 (>15%) and lowest for Δ2-Δ6 (<9%) (Supplementary Fig. S2B and Fig. 1E, middle). The thermal denaturation profiles determined by DSF were characterized by a broad peak with a small shoulder for the six longest constructs (A3-Δ2), revealing a principal thermal transition (melting temperature, T_m_) of ~65 °C (Supplementary Fig. S3 and Fig. 1E, bottom). In contrast, T_m_ values were significantly reduced and highly variable for the shorter constructs. Two of these (Δ5 and Δ8) exhibited broad profiles resembling those of the longer constructs, with T_m_ values of ~60 °C. The remaining constructs (Δ3-Δ4 and Δ6-Δ7) displayed profiles with two distinct peaks, characterized by a principal T_m_ value of 48–54 °C. Comparing these data with the T_m_ values of the isolated MBP and GS proteins (55 and 62 °C, respectively) reveals that the fusion of MBP with GS enhances subunit stability when the junction contains at least 12 residues (constructs A3-Δ2), whereas for shorter constructs (Δ3-Δ8) the degree of (de)stabilization depends on the precise junction length. Taken together, the above data indicate that the optimal construct for cryo-EM analysis is Δ2, which has the lowest R_h_ and is among the constructs with the lowest polydispersity and highest thermal stability. The second most promising construct appears to be Δ5, with a similar polydispersity index and R_h_ as MBP-GSΔ2 but a somewhat lower T_m_.

**Figure 2:**
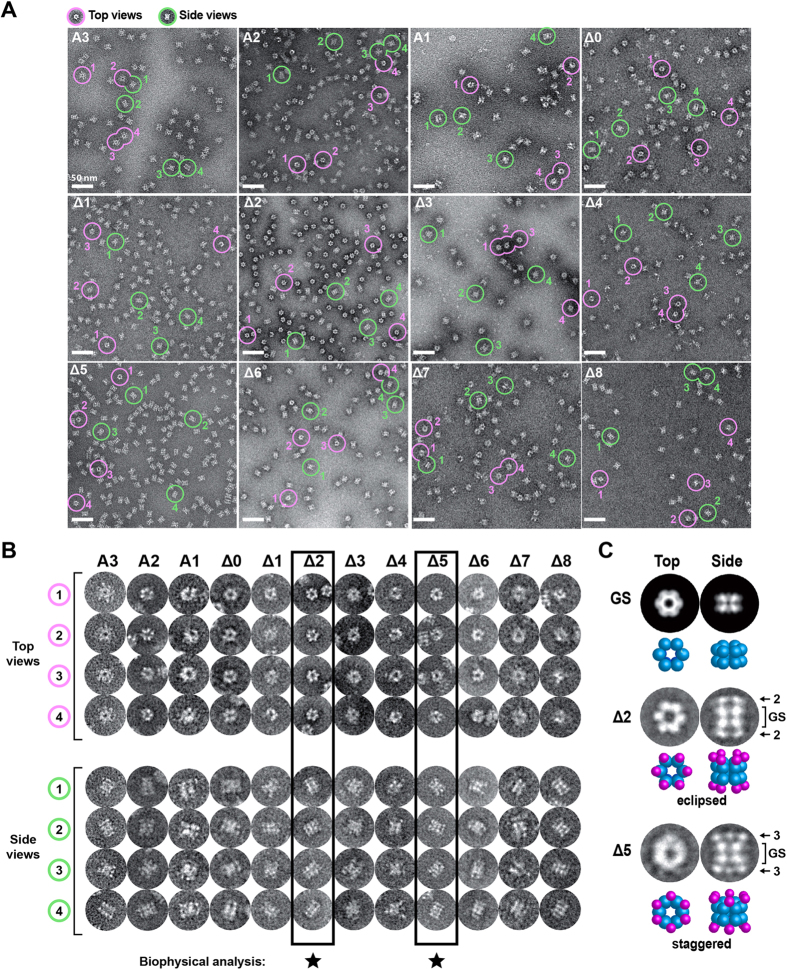
Negative-stain EM analysis of MBP-GS fusion proteins. (**A**) Electron micrographs of fusion proteins stained with sodium silicotungstate. Top and side views of particles are circled in magenta and green, respectively. Scale bar, 50 nm. (**B**) Magnified view of particles circled in (**A**). The two most promising constructs identified by biophysical analysis are boxed. (**C**) Class averages for top and side views of constructs Δ2 and Δ5 obtained by analysis of a small dataset (400 particles per construct). For comparison, the predicted projection of isolated GS is shown in the same orientation.

We next used negative-stain EM to assess the homogeneity of constructs A3-Δ8. All constructs yielded particles with recognizable top and side views (Fig. 2A). Top views presented ring-like shapes with different-sized protrusions that caused the outer diameter to vary between 14 and 20 nm (depending on the construct), compared to the 14 nm diameter observed for isolated GS particles (Supplementary Fig. S1A). Side views were characterized by four horizontal layers spanning a height of ~18 nm, compared to the two layers of height ~10 nm observed for the isolated GS dodecamer. These observations are consistent with chimeric particles having a central double ring of GS subunits sandwiched between two rings of MBP subunits, confirming the successful scaffolding of the monomeric target. Visual inspection revealed significant conformational heterogeneity for the majority of constructs, consistent with an irregular positioning of MBP subunits relative to the GS dodecamer (Fig. 2B). A notable exception was construct Δ2, which presented a highly regular and compact appearance, in agreement with the biophysical analysis. Construct Δ5 also appeared relatively homogeneous. Interestingly, 2D class averages obtained for top and side views of these two constructs revealed striking differences (Fig. 2C). In the top view, construct Δ2 displays a flower-like shape with pronounced 6-fold symmetry, whereas Δ5 has a smoother donut-shaped appearance. The side view for Δ2 is characterized by two distinct features within each MBP layer, whereas that of Δ5 shows three such features. These observations suggest a configuration in which the MBP and GS subunits of construct Δ2 are approximately aligned along the (6-fold) axial direction, such that adjacent rings of MBP and GS subunits stack with an eclipsed geometry. In contrast, the corresponding Δ5 subunits appear to be staggered, reflecting a rotational offset between adjacent MBP and GS subunit rings. These findings underscore how a minor change in the junction length can dramatically alter the relative positioning of MBP and GS subunits, thereby affecting the overall shape and homogeneity of the chimeric particle.

### Comparison of MBP-GS constructs Δ2 and Δ5 by cryo-EM

**Figure 3:**
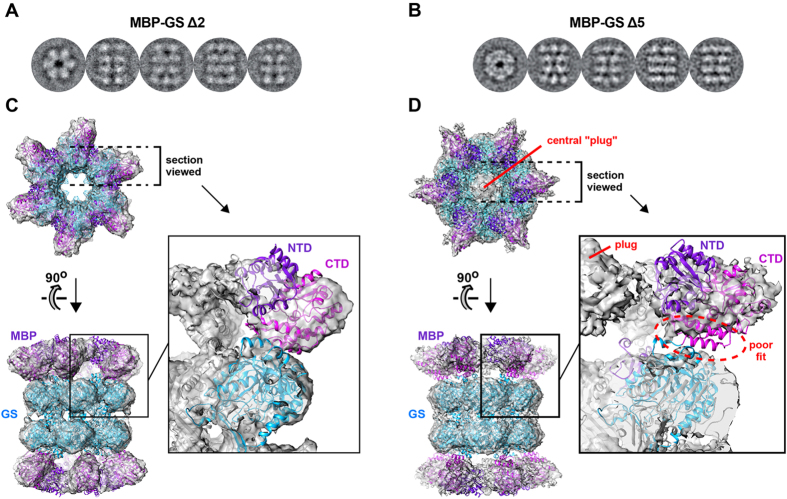
Initial cryo-EM analysis of MBP-GS constructs Δ2 and Δ5. (**A,B**) Representative class averages showing top and side views for MBP-GS constructs (**A**) Δ2 and (**B**) Δ5. (**C,D**) Fit of the MBP and GS crystal structures into the cryo-EM reconstructions obtained for construct (**C**) Δ2 and (**D**) Δ5. Both maps are countoured at 1.5 σ. The N- and C-terminal domains of MBP are labelled NTD and CTD and colored in purple and magenta, respectively. The GS subunit is in cyan. For construct Δ5, the central plug of density and the MBP region that lies outside the map are indicated in red.

Based on the above results, we pursued cryo-EM analysis of MBP-GS constructs Δ2 and Δ5. To assess which of these was the more promising, we collected preliminary datasets on photographic film using an FEI Tecnai F30 Polara electron microscope. Raw images and class averages recapitulated the characteristic top and side views observed by negative-stain EM (Fig. 3A,B and Supplementary Fig. S4A,B). By imposing D_6_ symmetry, we obtained 3D reconstructions at an overall resolution (FSC = 0.143 criterion) of ~10 Å for construct Δ2 and ~15 Å for Δ5 (Supplementary Fig. S4C,D). For both reconstructions, the local resolution was higher for the central GS dodecamer than for the peripheral MBP subunits (Supplementary Fig. S4E,F). As suggested by negative-stain EM, the relative positioning of MBP and GS subunits differs dramatically between the two constructs: when viewed along the 6-fold, each MBP subunit is aligned with a neighbouring GS subunit in construct Δ2, but lies between two GS subunits in construct Δ5.

The crystal structure of the GS dodecamer fits well into the central region of both reconstructions, with most residues enclosed by the map and most of the map filled with atoms (Fig. 3C,D). In contrast, the MBP crystal structure fits markedly better into the reconstruction obtained for Δ2 than for Δ5. For Δ5, significant portions of MBP lie outside the map, including residues immediately adjacent to the GS subunit, unlike Δ2 for which continuous density is observed between the MBP and GS subunits. Moreover, the Δ5 reconstruction exhibits a central “plug” on the 6-fold axis which is isolated from neighbouring parts of the map and cannot be accounted for by MBP or GS residues. These map artefacts presumably arise because of flexibility within the Δ5 particle, leading to variable MBP subunit orientations that get averaged during the symmetry-imposed map calculation. Conversely, the improved quality of the MBP density observed for Δ2 suggests that the target subunit is more rigidly immobilized in this construct. These findings are consistent with the different geometries of the two particles: the eclipsed configuration of Δ2 stabilizes MBP subunits through contacts with adjacent GS subunits, whereas the staggered configuration of Δ5 leaves MBP subunits relatively unbuttressed. This difference in intersubunit contact also agrees with the enhanced thermal stability of construct Δ2 relative to Δ5 (Fig. 1E). These observations highlight the importance of a stable target-scaffold interface for accurate structure determination by cryo-EM and the usefulness of screening constructs for thermal stability. Given the above findings we abandoned construct Δ5 in favour of construct Δ2.

### Cryo-EM reconstruction of MBP at sub-nanometre resolution

**Figure 4:**
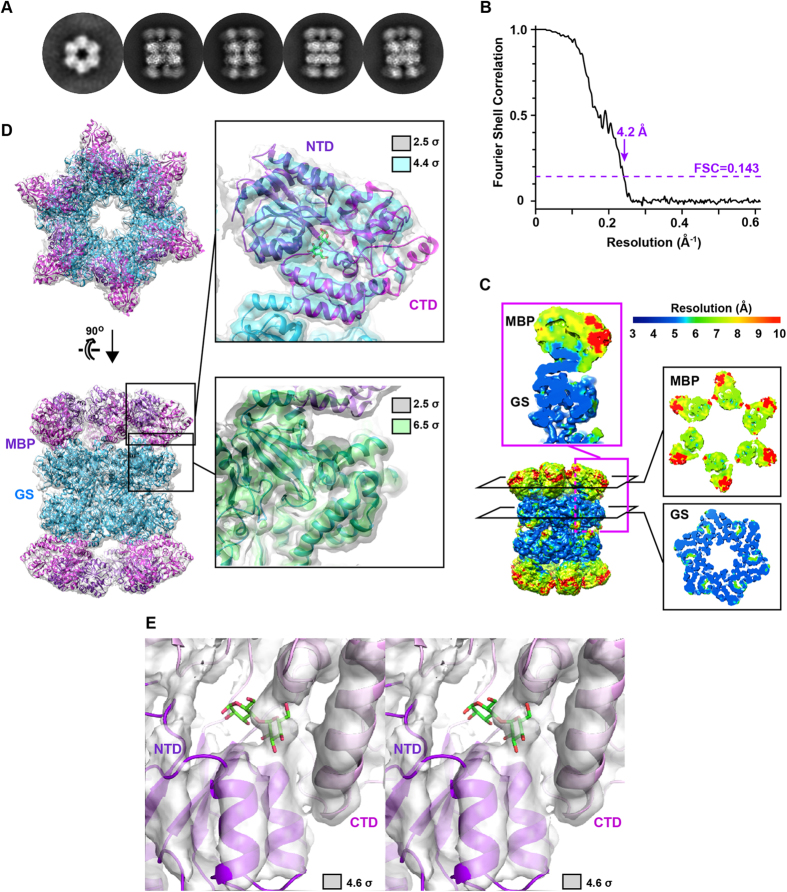
Final cryo-EM reconstruction of MBP-GS construct Δ2. (**A**) Representative class averages showing top and side views. (**B**) FSC curve of the 3D reconstruction (calculated prior to masking). (**C**) Local resolution plotted onto the 3D reconstruction colored from blue (≤3 Å) to red (≥10 Å). Insets show different cross-sections of the MBP and GS subunit rings (boxed in black) or of an MBP-GS monomer (boxed in magenta). (**D**) Fit of the MBP and GS crystal structures into the 3D reconstruction. The N- and C-terminal domains of MBP (NTD, CTD) are in purple and magenta, respectively; the GS subunit is in cyan. Insets show the cryo-EM map covering one MBP or GS subunit. The 3D reconstruction is displayed at two different isosurface levels for each domain. Contour levels are 2.5 σ (gray), 4.2 σ (cyan) and 6.5 σ (green). (**E**) Stereo view showing the active site of MBP, with maltose indicated in stick representation. The map is contoured at 4.6 σ.

**Figure 5:**
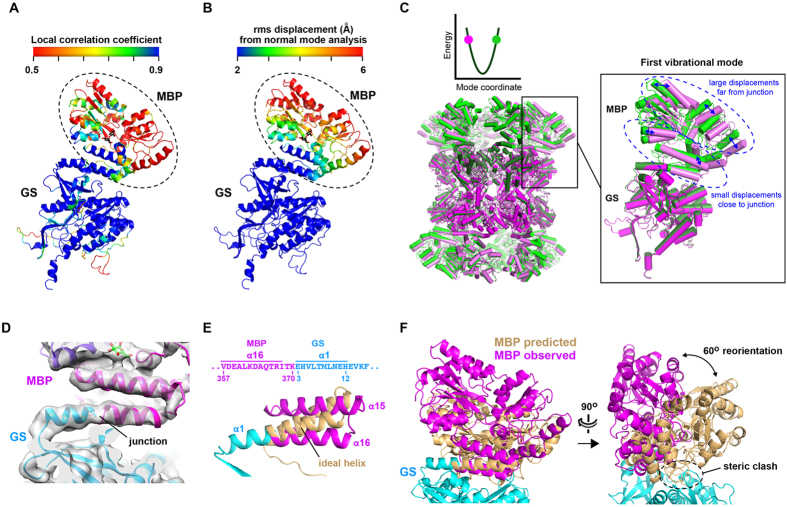
Mobility and orientation of the MBP subunit. (**A**) Plot showing the agreement between the 3D reconstruction and the MBP and GS crystal structures fitted into the map. Correlation coefficients between residues and the cryo-EM map were calculated at 8 Å resolution by program Phenix^34^ and colored from red (CC ≤ 0.5) to blue (CC ≥ 0.9). (**B**) Plot of residue mobility predicted by normal mode analysis. Rms displacements calculated by program ElNemo^59^ were scaled to have a mean value of 1 Å within the GS subunit and are plotted from blue (≤2 Å) to red (≥6 Å). (**C**) First vibrational normal mode of the MBP-GS Δ2 particle. Conformations are shown at phase values of +90^o^ (green) and −90^o^ (green) along the mode coordinate. *Inset.* Oscillation of the MBP subunit results in large displacements for residues distal from the junction and small displacements for proximal residues. Vibrational modes 1–6 are presented in Supplementary Video S3. (**D**) Cryo-EM map of the MBP-GS junction region. (**E**) Distortion of the helical junction region. The C-terminal helix of MBP fitted into the cryo-EM map (α16, magenta) deviates significantly from the orientation predicted by superimposing a helix with ideal geometry (beige) onto the N-terminal helix of GS (α1, cyan). (**F**) Orientation of the MBP subunit fitted into the cryo-EM map (magenta) compared to that predicted if the junction region had ideal helical geometry (beige).

To obtain a higher resolution cryo-EM structure for MBP-GS construct Δ2 we collected a larger dataset, used an electron-counting direct detection camera and performed 3D classification (Supplementary Fig. S5A–C and Fig. 4A). This led to a new reconstruction with an overall resolution of 4.2 Å (FSC = 0.143 criterion) and a local resolution that varied from ~4 Å within the GS subunit to between 6 and 10 Å within the MBP subunit (Fig. 4B,C). This map agreed well with the GS crystal structure, allowing all secondary structure elements and certain large side chains to be visualized (Fig. 4D, Supplementary Fig. S5D and Supplementary Video S1). A good fit was also observed for MBP: the N- and C-terminal lobes enclosing the active site are clearly defined, several helical elements are well resolved, and the bound maltose ligand is partly visible (Fig. 4D,E and Supplementary Video S2). To better evaluate the accuracy of the reconstruction, we used program Phenix^34^ to calculate real-space correlation coefficients (RSCCs) between MBP and GS residues and the cryo-EM map. The resulting values generally mirrored the local resolution of the map, with the highest and lowest RSCCs associated with GS and MBP residues, respectively (Fig. 5A). Within MBP, the values were highest for residues close to the N-terminus of GS and decreased with increasing distance from this junction point. This fall-off in map quality is probably due to variations in the MBP subunit orientation relative to the scaffold caused by flexibility of the MBP-GS junction.

To verify this hypothesis, we performed normal mode analysis (NMA) of the MBP-GSΔ2 structure. NMA allows one to evaluate protein flexibility by decomposing large-scale motions such as domain and subunit movements into different vibrational modes and frequencies^35^,^36^,^37^. The lowest-energy vibrational modes are the most significant, with, typically, one or only a few such modes accounting for functionally relevant domain motions^38^,^39^,^40^. NMA confirmed that the MBP subunit within construct Δ2 is significantly more mobile than the GS scaffold: the predicted rms displacement of MBP residues is on average 4.5 times higher than for GS (Fig. 5B). Within MBP, these displacements are small for residues close to the junction and large for more distal residues. This is illustrated by the lowest-energy vibrational modes, which reveal how the MBP subunit can “wobble” about the junction point (Fig. 5C and Supplementary Video S3). Such flexibility arises because of the small interface between MBP and GS subunits compared to the large interfaces shared between GS subunits. NMA thus rationalizes why the resolution and quality of the 3D reconstruction are higher for the scaffold than for the target, and higher for MBP residues close to the junction than for those farther away. These findings raise the possibility of improving the target map resolution by introducing mutations on the GS surface that stabilize the interface with MBP. Indeed, the mutation of interfacial residues has previously been used with success to enhance the rigidity and conformational homogeneity of a chimeric nanocage^41^. The resolution of the MBP region of the map might feasibly also be improved by applying a localized 3D reconstruction strategy to correct for the variability in subunit orientation^42^.

Inspection of the junction region shows a continuous tube of density between the C-terminus of MBP and the N-terminus of GS, suggesting the formation of a single helix spanning these subunits (Fig. 5D). Strikingly, the observed position of the C-terminal MBP helix deviates significantly from that predicted by extending the N-terminal GS helix with ideal geometry (Fig. 5E). This deviation corresponds to a 60° rotation of the MBP subunit relative to the position expected if the junction formed an ideal helix (Fig. 5F). This rotation is considerably larger than the 7–35° deviations reported in a recent study in which two protein domains with helical termini were joined via a helical linker^43^. The observed 60° distortion is required to relieve a severe steric clash which an ideally helical junction would induce between MBP and GS subunits (Fig. 5F). Distortion of the intersubunit helix relieves the steric overlap and permits favourable contacts between MBP and GS, which stabilize the MBP orientation and thereby extend the D_6_ symmetry from the scaffold to the target. These observations underscore the value of systematically screening different junction lengths to optimize the construct via a trial-and-error process, as the structure and rigidity of the MBP-GSΔ2 particle would have been difficult to predict otherwise.

## Discussion

This study set out to explore the feasibility of scaffolding a small monomeric protein (<100 kDa) onto a homo-oligomeric structure to enable cryo-EM analysis. By fusing monomeric MBP to dodecameric GS and progressively deleting residues in the junction, we generated a series of MBP-GS constructs that assembled as chimeric particles sufficiently large for cryo-EM analysis. Biophysical assays combined with negative-stain EM identified the two most promising MBP-GS constructs (Δ2 and Δ5). For one construct (Δ2), the 12 MBP subunits within the particle adopt the same orientation relative to the GS subunits to which they are fused. This scaffold-mediated “symmetrization” of the target yielded a single-particle reconstruction that revealed the structure of MBP at sub-nanometre resolution, thus providing proof of concept.

The use of a homo-oligomeric scaffold to induce target symmetrization has several advantages. First, the large mass of the chimeric protein facilitates the identification and orientation determination of particles. Second, the known structure of the scaffold provides a useful validation of the 3D reconstruction. Third, the high symmetry reduces the number of particles required for data analysis. Also, compared to a large monomeric scaffold, a homo-oligomeric scaffold has the added advantage that the conformational homogeneity of fusion constructs can be readily evaluated by visual inspection of negative-stain EM images, where variability in the target-scaffold interface is recognizable as a lack of symmetry in the overall particle. The success of the scaffolding approach to enable cryo-EM analysis of MBP raises the prospect of applying this strategy to monomeric targets which are resistant to NMR or crystallographic analysis, e.g., proteins above a certain size (~50 kDa) which fail to yield well-diffracting crystals. Even a low-resolution cryo-EM structure of such proteins could give important functional insights or provide clues ultimately enabling crystallographic or NMR analysis. Inspection of our MBP-GSΔ2 structure suggests that the GS scaffold can accommodate targets significantly larger than MBP, as neighbouring MBP subunits do not interact but are well separated within the Δ2 particle (Supplementary Fig. S5E). Indeed, a larger target able to interact with its neighbours might be advantageous, as the additional contacts could help stabilize the target orientation within the particle.

Future studies are required to evaluate additional homo-oligomeric proteins as potentially useful scaffolds together with additional, diverse monomeric targets. In particular, whether scaffold-induced symmetrization can successfully be applied to a target lacking an N- or C-terminal helix remains to be determined. However, the scaffolding strategy remains potentially useful even if limited only to proteins bearing a terminal helix, as numerous proteins fall within this category. Interestingly, a recent study reported the stabilization of a helical junction through the use of a bifunctional, cysteine-specific cross-linking reagent^44^. Such a cross-linking strategy could conceivably improve the reconstructions obtained for both MBP-GS constructs Δ2 and Δ5, but might also be used to rigidify a non-helical junction. In practice, for any given target it may be necessary to screen several scaffolds of different size and shape before obtaining a suitably rigid chimeric particle. The ability to screen constructs by biophysical analysis and negative-stain EM significantly reduces the amount of cryo-EM data acquisition and analysis required, thereby saving on costly, labour-intensive steps.

In conclusion, we have shown that fusion to a dodecameric protein followed by junction optimization allows the structure of a 40 kDa monomer to be determined at sub-nanometre resolution by cryo-EM. This result demonstrates that homo-oligomeric scaffolding can feasibly be used to expand the range of molecules amenable to cryo-EM analysis to include “ordinary-sized” monomeric proteins, paving the way for application of this strategy to more challenging structural targets.

## Methods

### Purification of proteins

#### GS

GS from *E. coli* was expressed as an N-terminally His-tagged protein from a pETM-11 plasmid. Transformed *E. coli* BL21 DE3 cells were grown in Luria-Bertani broth (LB) containing 50 μg/mL kanamycin at 37 °C until an OD_600_ of 1.0 and protein expression was induced with 1 mM IPTG overnight at 20 °C. Harvested cells were lysed in lysis buffer (50 mM Tris pH 8, 200 mM NaCl, 20 mM imidazole, 5 mM β-mercaptoethanol, 10 mM MgCl_2_) in the presence of lysozyme (1 mg/mL) and protease inhibitors by sonication at 4 °C and centrifuged at 40,000 g for 20 min. The clarified lysate was applied to a Ni-NTA resin (500 μL/L culture) and washed with lysis buffer. Proteins were eluted in the same buffer containing 500 mM imidazole, concentrated by ultrafiltration and purified on a Superose 6, 10/300 GL column (GE Healthcare) in 50 mM TRIS pH 8, 150 mM NaCl. All buffers contained 10 mM MgCl_2_ to stabilize the dodecameric state of GS^45^.

#### MBP-GS fusion constructs

MBP-GS fusion constructs were cloned in pETM-11 vectors (EMBL) by restriction ligation between NcoI/KpnI sites and deletions performed using a QuikChange kit (Agilent). Transformed *E. coli* BL21 DE3 cells were grown in LB containing 50 μg/mL kanamycin at 37 °C until an OD_600_ of 1.0 and protein expression was induced with 1 mM IPTG overnight at 20 °C. Cells were centrifuged at 5000 g for 20 min, resuspended in lysis buffer (50 mM Tris pH 8, 200 mM NaCl, 5 mM MgCl_2_, 5 mM β-mercaptoethanol) in the presence of lysozyme (1 g/L) and protease inhibitors, lysed by sonication at 4 °C and then centrifuged at 40,000 g for 20 min. The clarified lysate was applied to an amylose resin (500 μL/L culture) and washed with lysis buffer. Proteins were eluted with the same buffer containing 10 mM maltose and further purified by gel filtration using a Superose 6, 10/300 GL column (GE Healthcare) in 50 mM TRIS pH 8, 150 mM NaCl, 5 mM MgCl_2_, 10 mM maltose.

### Biophysical analysis

#### Native PAGE

5 μg of each MBP-GS fusion were loaded on a 4% polyacrylamide gel and a voltage of 150 V was applied for 7 h at 4 °C using 150 mM TRIS/glycine pH 8.8 as the running buffer. Proteins were visualized by Coomassie blue staining.

#### Differential scanning fluorimetry

DSF was performed on MBP-GS constructs at a final concentration of 5 μM in white 96-well plates in an RT-PCR machine (Bio-Rad CFX96), as described^46^. Each well (20 μl) contained SyproOrange Dye (Sigma-Aldrich) diluted 5000x. The plate temperature was ramped from 20 to 99 °C with a 0.5 °C temperature increment. The dye was excited at 483 nm and fluorescence intensity detected at 568 nm. T_m_ values were calculated as the temperature at which the first derivative of thermograms (dF/dT) displayed a maximum using the integrated Bio-Rad software CFX-manager 2.1.

#### Dynamic light scattering

DLS experiments were performed on 40 μM MBP-GS fusion constructs in 50 μL cuvettes using a DynaPro Nanostar instrument (Wyatt Technology) at 25 °C. A He-Ne laser illuminated the solution at λ = 632.8 nm and the time-dependent (every 5 μs) fluctuations in scattering intensity were recorded every 5 μs. The polydispersity index was calculated using the integrated Dynamics 7 software.

### Negative stain EM

A volume of 4 μL of each MBP-GS fusion protein (20 μg/mL) was applied to the clean side of carbon on mica (carbon/mica interface). The carbon layer was subsequently floated onto a 2% sodium silicotungstate solution (pH 7.4), recovered with a 400 mesh copper grid (Agar) and air dried for 10 min. Micrographs were taken under low-dose conditions (exposing for 1 s at an electron dose of 30 e−/Å^2^) on a Philips CM12 microscope operated at 120 kV. Images were recorded at a nominal magnification of 22000X with a defocus of −1.5 μm on a Gatan Orius 1000 CCD camera, corresponding to a pixel size of 3.24 Å/pixel on the specimen.

### Cryo-EM analysis

#### Initial reconstructions of MBP-GSΔ2 and Δ5

Aliquots (4 μL) of purified MBP-GS constructs Δ2 and Δ5 (0.5 g/L) were applied to Quantifoil holey carbon grids (Cu/Rh, 400 mesh, 1.2/1.3 μm) at 100% humidity and 20 °C, blotted with filter paper for 2 s and vitrified in liquid ethane using a Mark IV Vitrobot (FEI). Data were recorded on photographic film using a Tecnai F30 Polara electron microscope (FEI) operated at 300 kV at a nominal magnification of 39,000X. Samples were exposed for 1 s for a total dose of 20–25 e−/Å^2^. Micrographs were selected by checking their power spectrum and scanned on a Zeiss scanner (Photoscan TD) at a step size of 7 μm (1.8 Å on the sample scale). The CTF of scanned micrographs was determined using CTFFIND3^47^ and corrected using the bctf routine in Bsoft^48^. Particles were selected semi-automatically in boxer (EMAN)^49^, yielding datasets of 16,025 and 8,797 particles (corresponding to ~7400 and ~5000 side views) for MBP-GS constructs Δ2 and Δ5, respectively. Datasets were normalized in IMAGIC^50^. Reference models were generated by angular reconstitution using IMAGIC and volumes refined by projection matching using SPIDER^51^. As a control to check for possible model bias, we generated an alternative *ab initio* reference model using RIco^52^, which uses symmetry adapted functions. Volumes derived from the two independent reference structures converged to similar reconstructions. The overall resolution was estimated by dividing the datasets in two halves and calculating the normalized Fourier shell correlation in SPIDER^51^.

#### Final reconstruction of MBP-GSΔ2

Grids vitrified as described above were examined using a Tecnai F30 Polara electron microscope (FEI) operated at 300 kV. Images were recorded manually using a K2 Summit direct electron detector (Gatan) in super resolution counting mode at a nominal magnification of 23,000X, which corresponds to a final pixel size of 0.8155 Å. (Data collection and processing parameters are summarized in Table S1.) Exposures were recorded with defocus values ranging from 1.0 to 3.5 μm. For each movie, a total of 40 frames were collected over 6 s, with a total dose of 25 electrons/Å^2^. For the initial processing, the 40 frames of each movie were aligned using MotionCorr^53^ and combined to generate a single averaged image. CTF parameters were determined using CTFFIND3^47^. A total of 39,167 particles selected from 165 averaged images were subjected to 2D classification in RELION^54^, leading to a cleaned dataset composed of 29,976 particles. This dataset was subjected to 3D classification (5 classes) using an angular step size of 7.5° and a reference model derived from the previous cryo-EM model (obtained from the dataset collected on film) by applying a low-pass filter to 30 Å and imposing D_6_ symmetry. The two most resolved 3D classes (~10 Å in resolution) were combined to yield a dataset of 13,847 particles, which were subjected to 3D refinement in RELION to yield a D_6_ symmetry-constrained map at 6.2 Å resolution as estimated by gold standard FSC in RELION (FSC = 0.143). Movie processing implemented in RELION improved the overall resolution to 4.2 Å (FSC = 0.143). Local resolution was determined with ResMap^55^ and confirmed by fitting and visual inspection. Fitting of the MBP and GS crystal structures was performed using Chimera^56^. The agreement between the fitted structures and the cryo-EM map was evaluated by calculating real-space correlation coefficients (RSCCs) at 8 Å resolution using Phenix^34^. RSCCs were averaged over a sliding window of 5 residues, assigned to the B-factor column of the coordinate file and plotted using PyMOL. Software used for structure calculations was compiled by SBGrid^57^.

### Normal mode analysis

Trajectories for the vibrational normal modes shown in Fig. 5C and Supplementary Video S3 were calculated for the structure of the MBP-GSΔ2 particle using the NOMAD-Ref web server^58^, which employs an elastic network model (ENM)^36^. All protein atoms of the dodecameric particle were included in the calculation, with the ENM cutoff for mode calculation, the distance weight parameter for the elastic constant, and the average rmsd of trajectories set to the default values of 5, 10 and 1 Å, respectively. Temperature (B) factors were determined by ElNemo^59^ by analysis of the first 100 vibrational modes calculated for a Cα model of the MBP-GSΔ2 dodecamer using the default settings for the amplitude range (-dqmin = dqmax = 100) and increment (dqstep = 20). B factors were scaled to have a mean value of 26.3 Å^2^ (corresponding to an rms displacement of 1 Å) within the GS subunit and converted to rms displacements (<R>) using the relation B = (8π^2^/3)<R>^2^.

## Additional Information

**Accession codes:** The cryo-EM structure and atomic coordinates of construct MBP-GSΔ 2 are available from the Electron Microscopy Data Bank and Protein Data Bank under accession codes EMD-4039 and 5LDF, respectively. 

**How to cite this article**: Coscia, F. *et al*. Fusion to a homo-oligomeric scaffold allows cryo-EM analysis of a small protein. *Sci. Rep.* **6**, 30909; doi: 10.1038/srep30909 (2016).

## Supplementary Material

Supplementary Information

Supplementary Video S1

Supplementary Video S2

Supplementary Video S3
