# Supplementary material for: Fusion to a homo-oligomeric scaffold allows cryo-EM analysis of a small protein
Source: Sci Rep. 2016 Aug 3;6:30909. doi: 10.1038/srep30909 (PMC4971460; doi:10.1038/srep30909)
Supplement: Supplementary Information [file srep30909-s1.pdf]

Supplementary Information

Fusion to a homo-oligomeric scaffold allows cryo-EM analysis of a small protein

Francesca Coscia, Leandro F. Estrozi, Fabienne Hans, Hélène Malet, Marjolaine Noirclerc-Savoye,
Guy Schoehn, Carlo Petosa

Table S1

Figures S1-S5

Videos S1-S3

Supplementary Methods

Supplementary References

Supplementary Table S1. Cryo-EM data collection and processing parameters for MBP-GSΔ2.

Data collection	
Microscope model	FEI Tecnai F30 Polara
Electron source	Field Emission Gun
Acceleration voltage	300 kV
Nominal defocus: minimum - maximum	1.0 - 3.5 μm
Alignment Procedure	Coma free
Detector	Gatan K2 Summit (4k x 4k)
Detector mode	Super-resolution
Movie frames per image	40
Range of movie frames used for the reconstruction	1-40
Average electron dose per image	25 e^-/A^2
Average exposure time per image	6 s
Number of images used	165
Reconstruction	
Particles selected at start of image processing	39,167
Particle selection software	EMAN[49], Relion[54]
CTF correction	Phase flipping
Software used to determine CTF	CTFFIND3[47]
Software used to apply CTF correction	bctf routine in Bsoft[48]
Startup model	IMAGIC[50], RIco[52]
Initial angle assignment	Angular reconstitution in IMAGIC[50]
Final angle assignment	Projection matching
Type of classification	3D
Number of classes	5
Number of particles used in final reconstruction	13,847
Resolution	4.2 Å
Resolution method	FSC 0.143 cut-off
Type of FSC	Even/odd maps refined independently (gold standard)
Point group symmetry	D6
Final reconstruction software	Relion[54]
EMDB accession ID	EMD-4039
Fitting of atomic models	
Fitting protocol	Rigid body fitting using Chimera[56]
Model used for maltose-binding protein (MBP)	PDB 1ANF
Model used for glutamine synthetase (GS)	PDB 1F52 (chains A-L as single rigid body)
Cross-correlation coefficient at 6.2 Å resolution	
MBP	0.8288
GS (dodecamer)	0.8830
PDB accession ID of MBP-GSΔ2	5LDF

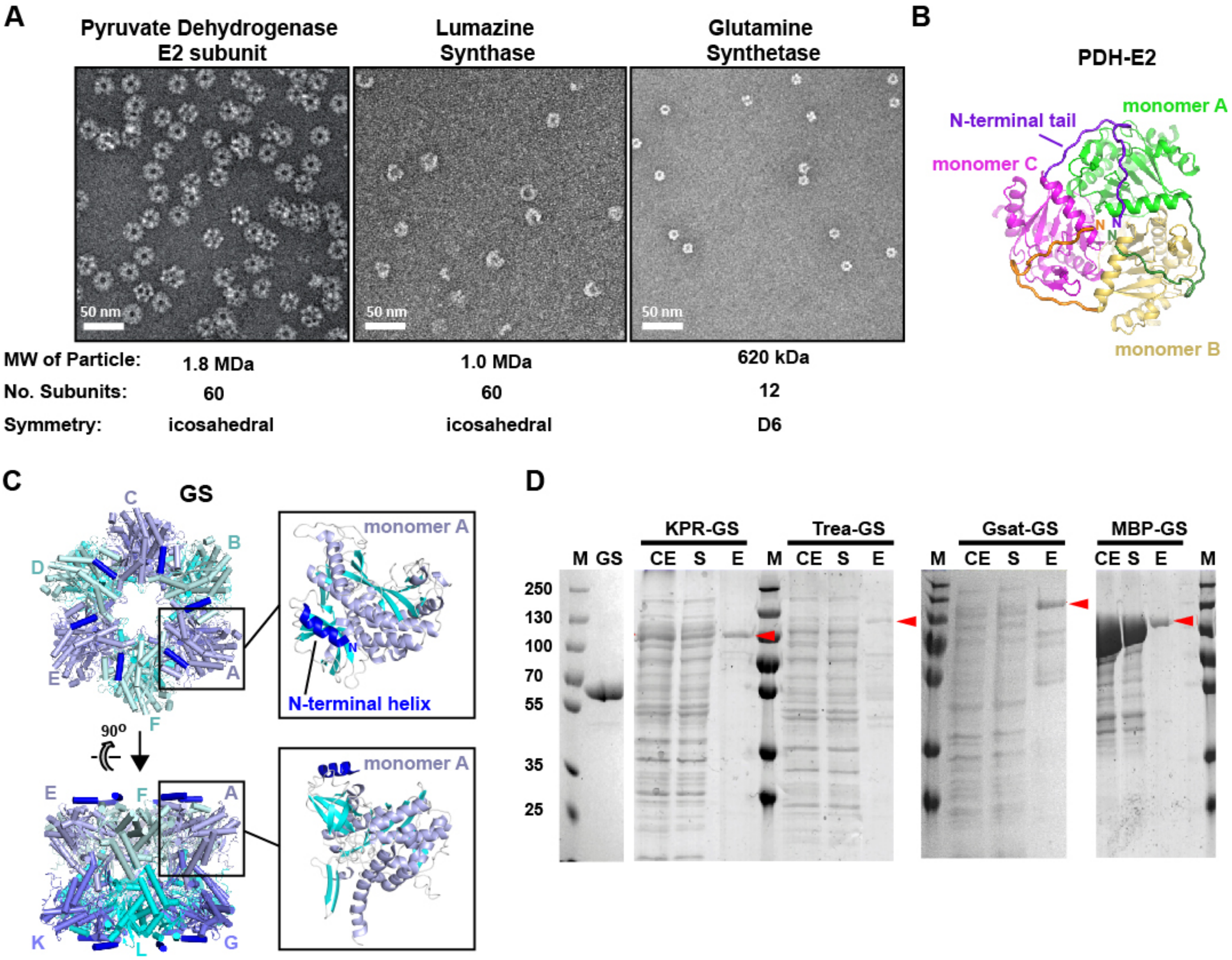

Supplementary Figure S1. Scaffold and target selection.

(A) Negative-stain EM of scaffold candidates. Three proteins were examined: lumazine synthase (LS), the E2 subunit of pyruvate dehydrogenase (PDH-E2), and glutamine synthetase (GS). Recombinantly expressed and purified proteins were stained with sodium silicotungstate. Whereas PDH-E2 and GS yielded homogeneous particles, LS particles showed considerable irregularity in their size and shape, leading us to discard LS as a possible scaffold.

(B) Crystal structure of PDH-E2. A trimer of PDH-E2 subunits within the icosahedral particle is shown (PDB entry 1B5S). The 20 N-terminal residues of each monomer interact with a neighbouring monomer within the trimer. These residues are believed to detach from the E2 surface when associated with the E1 and E3 subunits within the PDH holo-complex (Milne et al., 2006). A similar detachment occurring upon fusion to a heterologous protein would frustrate attempts to generate a rigid chimeric particle, leading us to discard PDH-E2 as our choice of scaffold.

(C) Crystal structure of dodecameric glutamine synthetase (GS) from *Salmonella typhimurium* (PDB entry 1F52). The *E. coli* GS used in the present study is expected to have the same structure (97% sequence identity). The N-terminal helix is highlighted in blue. Compared to a random coil, a helical conformation at the scaffold N-terminus would reduce the flexibility of the junction and potentially form a helical bridge with the target subunit, thereby enhancing the rigidity of the chimeric particle. These considerations led us to choose GS as the scaffold for subsequent constructs.

(D) Expression tests of bacterial proteins fused to GS. To identify a convenient target protein we searched the PDB for monomeric proteins that were "medium-sized" (35-60 kDa) and likely to be properly folded when fused to GS. We selected four bacterial proteins: Ketopanthoate reductase (KPR, 37 kDa), trehalase (Trea, 60 kDa), glutamine synthetase adenylyltransferase (Gsat, 50 kDa) and maltose binding protein (MBP, 41 kDa). These proteins were fused to the N-terminus of GS via an 18-residue linker sequence with high helix-forming propensity, AMAKALEAQKQKEQRQAA (derived from ribosomal protein L9 with the aim of restricting flexibility between the fused subunits). Fusion constructs were expressed in *E. coli* and purified by Ni-NTA affinity chromatography. Samples were analyzed by SDS-PAGE. All four candidate targets yielded soluble fusion proteins of the expected size (red arrows). MBP yielded the highest expression level and hence was selected as the target so as to facilitate the expression and purification of subsequent fusion proteins derived from this construct. CE, crude extract; S, soluble fraction (clarified lysate); E, elution fraction from the NiNTA affinity step.

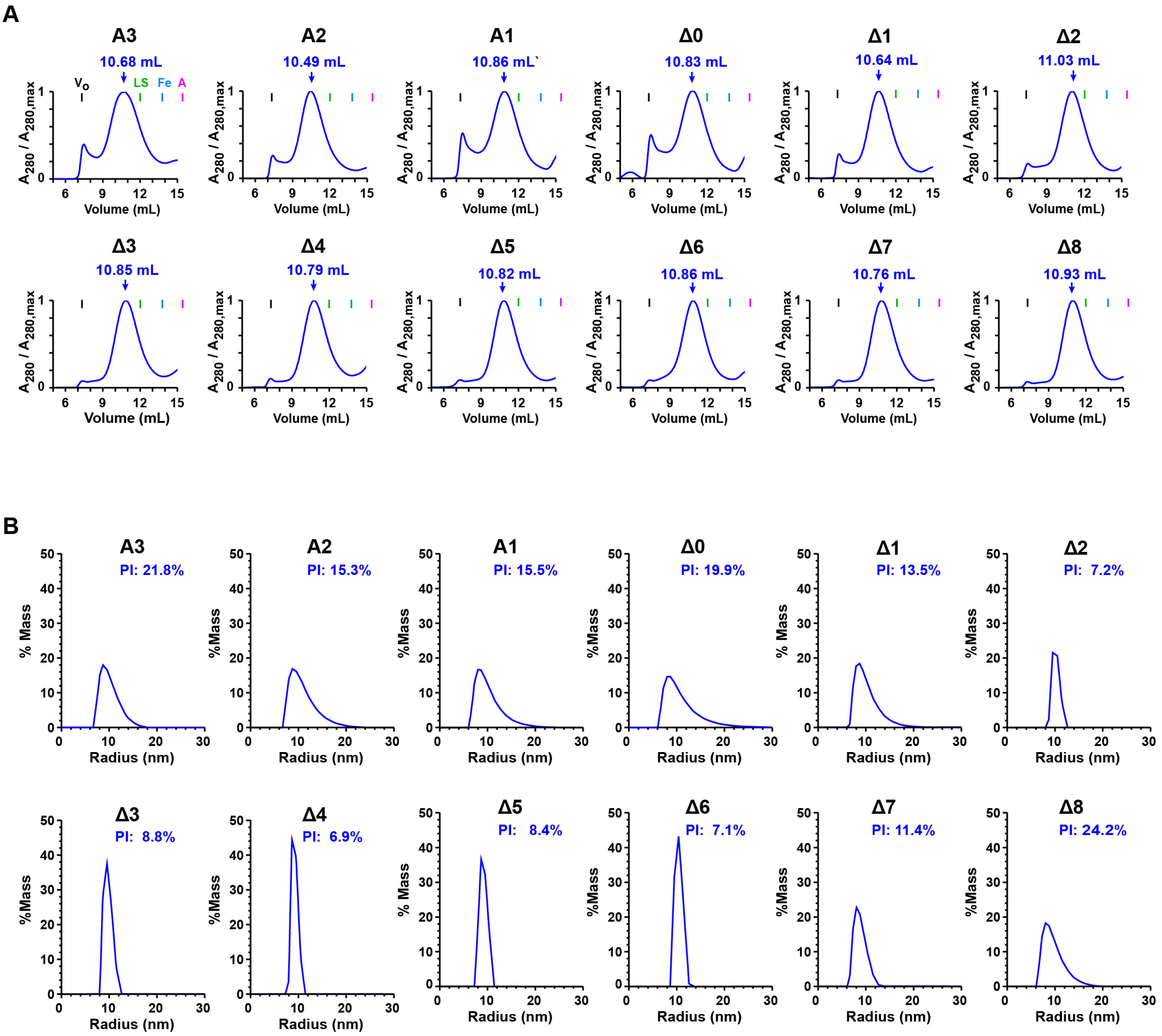

Supplementary Figure S2. Size exclusion chromatography (SEC) and dynamic light scattering (DLS) analysis of MBP-GS fusion constructs A3-Δ8.

(A) SEC analysis. Constructs were analyzed on a Superose 6 10/300 GL column (GE Healthcare). Elution volumes for fusion constructs are indicated by blue arrows. The void volume (V_o) is indicated by a black mark, and the elution volumes of proteins used to calibrate the column - lumazine synthase (LS, 1 MDa), ferritin (Fe, 444 kDa) and aldolase (A, 158 kDa) - are shown by green, blue and magenta marks, respectively.

(B) DLS analysis. The polydispersity index (PI) of each construct is indicated.

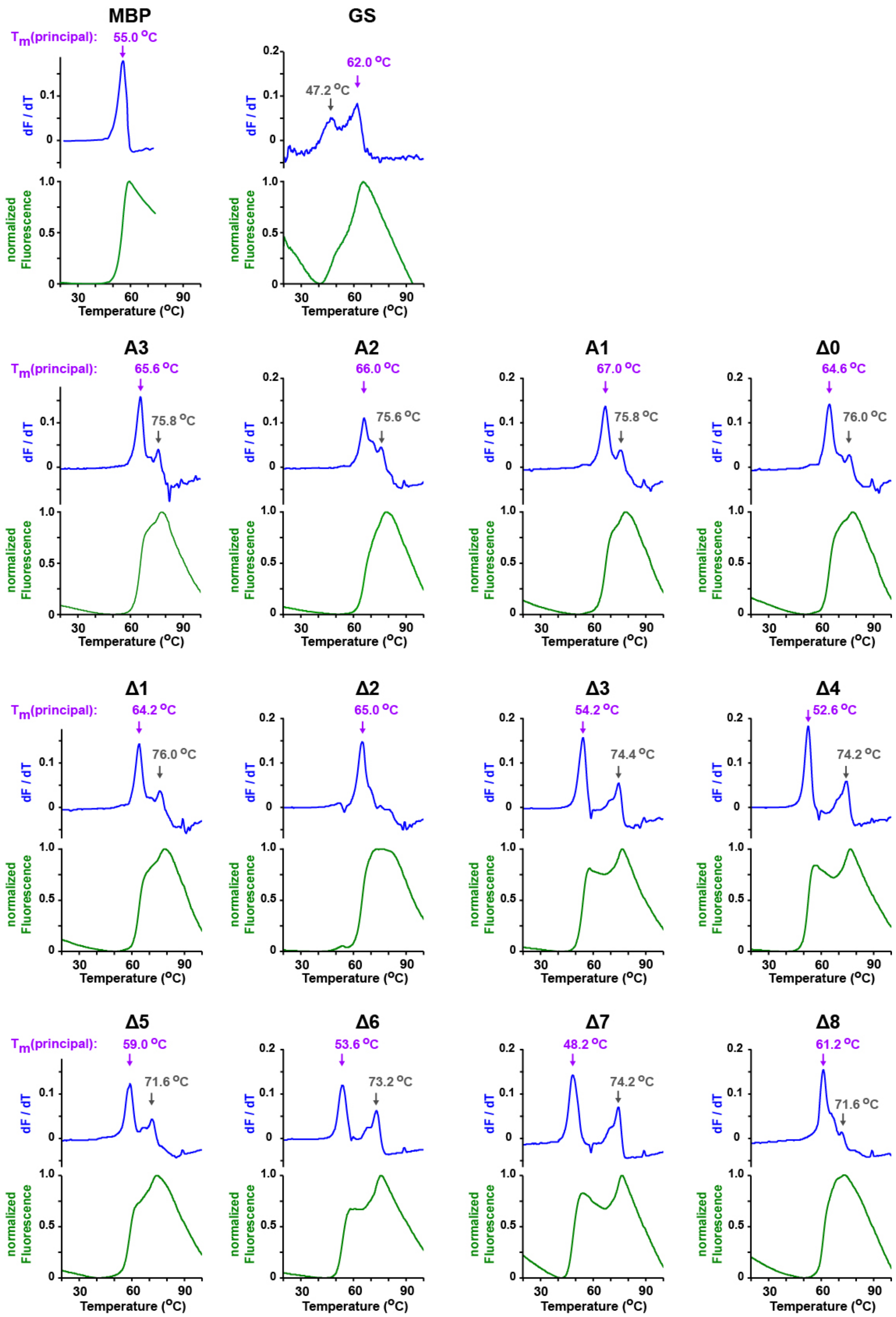

Supplementary Figure S3. Differential scanning fluorimetry analysis of MBP, GS and MBP-GS constructs A3-Δ8.

Thermal denaturation profiles and their first derivatives are shown in green and blue, respectively. The principal T_m derived from the major inflection point is indicated in magenta. The denaturation profile for isolated MBP revealed a single transition at 55 °C, while that for isolated GS displayed a principal transition at 62 °C and a smaller one at 47 °C. This is consistent with a previous report, which attributes the smaller transition to partial unfolding of the dodecamer, and the larger transition to a loss of secondary structure [60].

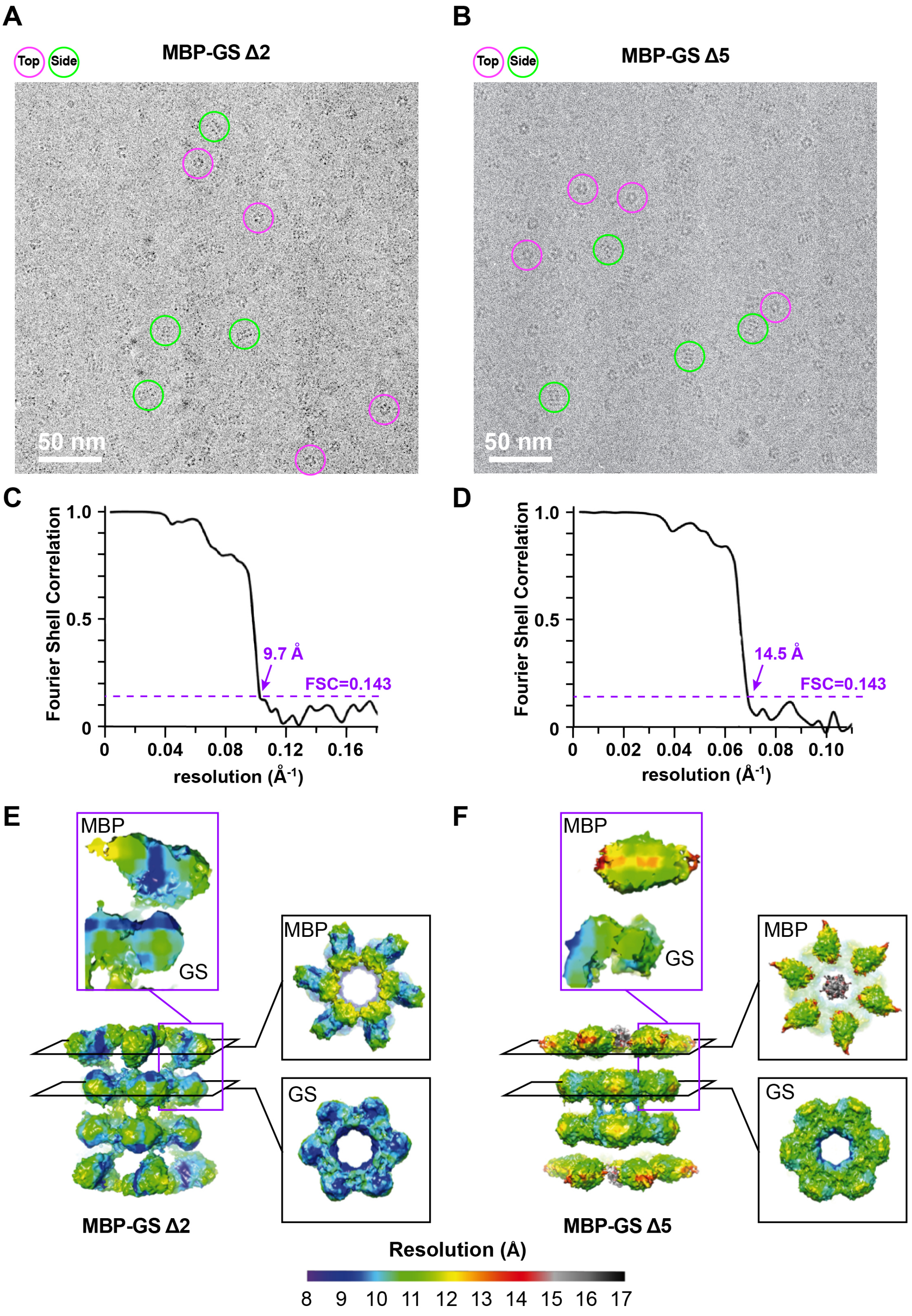

Supplementary Figure S4. Preliminary cryo-EM analysis of constructs MBP-GSΔ2 and MBP-GSΔ5.

(A, B) Raw low-dose images of the frozen hydrated constructs (A) MBP-GSΔ2 and (B) MBP-GSΔ5 embedded in a thin layer of vitrified ice. Top and side views of particles are circled in magenta and green, respectively. Images were collected on film .

(C,D) FSC curves of the 3D reconstructions for MBP-GS construct (C) Δ2 and (D) Δ5. The resolution corresponding to FSC = 0.143 (calculated prior to masking) is indicated.

(E, F) Local resolution determined using Blocres[61] plotted onto the 3D reconstruction of constructs (E) Δ2 and (F) Δ5, colored from blue (≤8 Å) to red (15 Å) and from light gray (15 Å) to black (≥17 Å). Insets show different cross-sections of the MBP and GS subunits (boxed in black) or of an MBP-GS monomer (boxed in magenta).

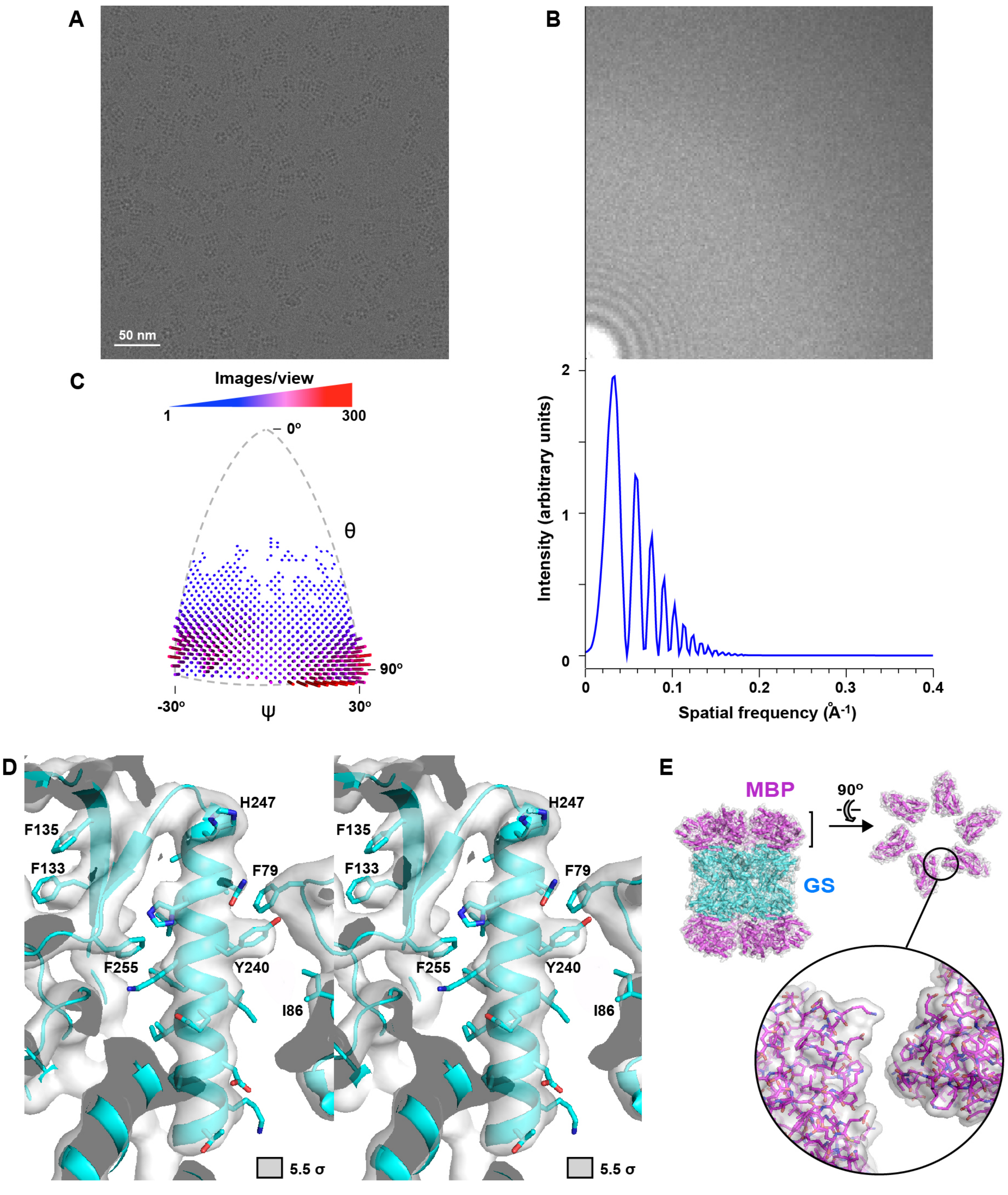

Supplementary Figure S5. Cryo-EM analysis of construct MBP-GSΔ2.

(A) A typical electron micrograph of construct MBP-GSΔ2 recorded with a K2 Summit GATAN camera in counting mode on a Tecnai POLARA instrument (FEI) operated at 300 kV.

(B) Portion of the power spectrum of the image in (A) (top) and estimated contrast transfer function (bottom), showing Thon rings that extend to approximately 1/5.5 $Å^{-1}$.

(C) Angular distribution of particle orientations used in the final reconstruction.

(D) Stereo view of the cryo-EM reconstruction showing part of the GS subunit. The view is centered on an α helix spanning residues 229-247 and shows neighbouring β strands spanning residues 132-137 and 252-254. Side chains are shown for the central helix and for bulky residues within neighbouring elements.

(E) Surface representation of MBP-GSΔ2 showing the lack of interactions between neighbouring MBP subunits.

Supplementary Videos

Video S1. Cryo-EM reconstruction of MBP-GS construct Δ2 showing the GS region of the map.

Video S2. Cryo-EM reconstruction of MBP-GS construct Δ2 showing the MBP region of the map.

Video S3. First six normal vibrational modes of the MBP-GSΔ2 particle, illustrating the greater mobility of the MBP subunits compared to the GS subunits.

Supplementary Methods

Purification of lumazine synthase (LS) and the E2 subunit of pyruvate dehydrogenase (PDH-E2). N-terminally His-tagged LS from *S. typhimurium* was expressed from a pET28c plasmid (obtained from S. Karthikeyan, Institute of Microbial Technology, Chandigarh) and purified as described by Kumar et al.[62] The gene encoding PDH-E2 from *B. stearothermophilus* was obtained from P. De Berardinis (Institute of Protein Biochemistry, Naples). PDH-E2 was expressed as an N-terminally His-tagged protein from a pETM-11 plasmid. Transformed *E.coli* BL21 DE3 cells were grown in Luria-Bertani broth (LB) containing 50 µg/mL kanamycin at 37 °C until an OD_{600} of 1.0 and protein expression was induced with 1 mM IPTG overnight at 20°C. Harvested cells were lysed in lysis buffer (50 mM Tris pH 8, 200 mM NaCl, 20 mM imidazole, 5 mM β-mercaptoethanol) in the presence of lysozyme (1 mg/mL) and protease inhibitors by sonication at 4 ºC and centrifuged at 40,000 g for 20 min. The clarified lysate was applied to an Ni-NTA resin (500 µL/L culture) and washed with lysis buffer. Proteins were eluted in the same buffer containing 500 mM imidazole, concentrated by ultrafiltration and purified on a Superose 6, 10/300 GL column (GE Healthcare) in 50 mM TRIS pH 8, 150 mM NaCl.

Supplementary References

60 Nosworthy, N. J. & Ginsburg, A. Thermal unfolding of dodecameric glutamine synthetase: inhibition of aggregation by urea. *Protein Sci* **6**, 2617-2623 (1997).

61 Cardone, G., Heymann, J. B. & Steven, A. C. One number does not fit all: mapping local variations in resolution in cryo-EM reconstructions. *J Struct Biol* **184**, 226-236 (2013).

62 Kumar, P., Singh, M. & Karthikeyan, S. Crystal structure analysis of icosahedral lumazine synthase from Salmonella typhimurium, an antibacterial drug target. *Acta Crystallogr D Biol Crystallogr* **67**, 131-139 (2011).
